# Retracted: Lentivirus-Mediated siRNA Targeting ER-*α* Inhibits Tumorigenesis and Induces Apoptosis in Hepatocarcinoma Cells

**DOI:** 10.1155/2020/3194276

**Published:** 2020-10-27

**Authors:** BioMed Research International

*BioMed Research International* has retracted the article titled “Lentivirus-Mediated siRNA Targeting ER-*α* Inhibits Tumorigenesis and Induces Apoptosis in Hepatocarcinoma Cells” [1] due to an error in the gene targeting.

It was raised to our attention [2] that the siRNA sequence said to target ER-*α* (estrogen receptor alpha), 5′ − GCCTTACAATGTACA GCAGAA − 3′, instead targets the similarly named but unrelated gene ERAL1 (Era Like 12S Mitochondrial RRNA Chaperone 1) [3].

Over 300 words, including the discussion of the use of siRNA, overlap with an article by other authors that was not cited, which also studied lentivirus-mediated RNA inference in hepatocarcinoma cells [4]. The file properties show the manuscript was edited by someone who is not one of the authors. The authors could not be contacted.
